# ALDH1A3 induces mesenchymal differentiation and serves as a predictor for survival in glioblastoma

**DOI:** 10.1038/s41419-018-1232-3

**Published:** 2018-12-11

**Authors:** Guanzhang Li, Yiming Li, Xing Liu, Zheng Wang, Chuanbao Zhang, Fan Wu, Haoyu Jiang, Wenlong Zhang, Zhaoshi Bao, Yongzhi Wang, Jinquan Cai, Liang Zhao, Ulf D. Kahlert, Tao Jiang, Wei Zhang

**Affiliations:** 1 Beijing Neurosurgical Institute, Capital Medical University, Beijing, China; 2 Chinese Glioma Genome Atlas Network(CGGA) and Asian Glioma Genome Atlas Network (AGGA), Beijing, China; 3 Department of Neurosurgery, Beijing Tiantan Hospital, Capital Medical University, Beijing, China; 4 Department of Neurosurgery, Beijing Huairou Hospital, Beijing, China; 5 Department of Neurosurgery, the Second Affiliated Hospital of Harbin Medical University, Harbin, China; 6 Department of Neurosurgery, University Medical Center Düsseldorf, Düsseldorf, Germany; 7 Center of Brain Tumor, Beijing Institute for Brain Disorders, Beijing, China; 8 China National Clinical Research Center for Neurological Diseases, Beijing, China

## Abstract

As aldehyde dehydrogenase (ALDH) is a novel stem cell marker, increasing studies have confirmed that high ALDH activity promotes tumorigenesis and progression in cancers. Some preliminary studies have found that ALDH1A3 may play an important role in glioma malignant progression, but so far there was no conclusive conclusion. The purpose of our study was to elucidate the mechanisms by which ALDH1A3 regulated in glioma and to provide practical tools for clinical application. Aldefluor, flow cytometry sorting and qRT-PCR were performed to verify the role of ALDH1A3 in ALDH activity maintenance. Transwell, immunofluorescence, glycolytic assays, and orthotopic xenograft models were used to explore ALDH1A3 bio-functions in GBM. LASSO-COX, COX survival analysis and Kaplan–Meier analysis were used to establish the prognostic evaluation system and predict postoperative chemotherapy sensitivity of GBMs. Our integrated study found that (1) ALDH1A3 associates with mesenchymal differentiation of GBM in Eastern and Western world patients. (2) ALDH1A3 plays a critical role in ALDH activity maintenance. (3) ALDH1A3 is an activator of mesenchymal transformation in GBM. (4) ALDH1A3-derived PMT markers’ molecular signature can predict 1-, 2-, and 3-year survival rates of GBMs precisely. In conclusion, ALDH1A3 was a major contributor to ALDH activity and a key driver in triggering mesenchymal transformation in GBM. ALDH1A3-based molecular classification scheme can help to improve guidance for prognosis forecasting and individualized treatment decision making for GBM patients.

## Introduction

Glioblastoma (GBM) is the most common and malignant primary brain cancer in adults^1^. GBMs are heterogeneous, infiltrating tumors characterized by high resistance levels to radiation and standard chemotherapy resulting to poor clinical outcome^2,3^. Accumulating evidence shows that glioma cells with stem cell properties, so-called glioma stem-like cells (GSCs), are responsible for tumor occurrence, progression and the emergence of therapy resistance^4–6^. Until now no targeted anti-GSCs therapy has been market-approved and strategies to efficiently and non-adversely eradicating GSCs are warranted.

Aldehyde dehydrogenases (ALDHs) are family members of one class of enzymes consisting of 19 different isoforms. Classically, those ALDHs are associated to metabolic functions being responsible for oxidizing aldehydes to carboxylic acids^7,8^. Recently, elevated ALDH activity has been found in various tumor types and functional studies revealed their profound role in promoting cancer stem-like cells^9–11^. Most recently, isoform ALDH1A3 prominently emerges as cancer stem-like cells target in neoplasms of the lung, bile duct, melanoma, prostate, and breast cancer^12–15^. ALDH1A3 has also been found to promote GSCs and attributed to be involved in the transdifferentiation to most malignant mesenchymal (MES) subtype of GBM^16^.

In our study, we identified ALDH1A3 to play cardinal decision-making roles in defining proneural or mesenchymal lineage of GBM as evidenced by various functional in vitro and in vivo studies. Moreover, we presented the hitherto largest assessment of ALDH1A3 in Western and Eastern world clinical databases of a total of 674 patients to verify the relationship between ALDH1A3 and MES GBM. Importantly, interrogating large data, we developed a practical molecular classification scheme centered on ALDH1A3 transcription to support its applicability for accurate prognosis determination in terms of patient overall survival.

## Materials and methods

### Samples and databases

This study was approved by the Beijing Tiantan Hospital institutional review board. Written consent was obtained from each patient. Only samples with >80% tumor cells were selected. Transcriptome microarray (Transcriptome Sequencing) and clinical data of glioma samples were from Chinese Glioma Genome Atlas (CGGA) generating with Agilent platform (Illumina Solexa). Overall survival was estimated from the date of diagnosis to the date of either death or last follow-up. Patient clinical characteristics are present in Table 1. The Cancer Genome Atlas (TCGA) database was obtained from TCGA portal sites (https://tcga-data.nci.nih.gov/tcga/tcgaDownload.jsp). The primary GSC transcriptome microarray database was downloaded from GSE67089 (https://www.ncbi.nlm.nih.gov/geo/).

**Table 1:** Clinical characteristics of GBMs in CGGA and TCGA databases

	CGGA database (GBM)	TCGA database (GBM)
Age		
Available (mean, range)	138 (47.1, 8–81)	536 (58.3, 11–89)
Not available	0	1
Gender		
Male	90	206
Female	48	329
Not available	0	2
KPS		
Available (mean, range)	82 (76.4, 20–100)	403 (77.1, 20–100)
Not available	56	134
Chemoradiotherapy		
Available	126	520
Not available	12	17
IDH1 status		
Mutant	32	31
Wild type	106	387
Not available	0	119
Transcriptome subtype		
Classical	47	145
Neural	11	83
Proneural	30	97
Mesenchymal	50	156
Not available	0	56
Overall survival time		
Available (mean, range)	138 (13.2, 1.1–69.1)	535 (15.3, 0.1–129.3)
Not available	0	2

*GBM* glioblastoma, *CGGA* Chinese Glioma Genome Atlas, *TCGA* The Cancer Genome Atlas, *KPS* Karnofsky performance score

### Cell culture

GBM cell lines LN229, U87, and U251 were obtained from the Institute of Biochemistry and Cell Biology, Chinese Academy of Science. All GBM cell lines were identified by STR Profiling and cultured in serum-free medium containing DMEM/F12 (Gibco) supplemented with B27 (Gibco), basic fibroblast growth factor (bFGF, 20 ng/mL), epidermal growth factor (EGF, 20 ng/mL), and heparin (2.5 mg/mL). Growth factors (bFGF and EGF) were added twice a week. GBM cell lines were enzymatically dissociated into single cells using Accutase (Sigma Aldrich) and thereafter routinely cultured in the serum-free medium every 4–6 days.

### Aldefluor assay and fluorescent-activated cell sorting

GBM cell lines were dissociated into single cells and followed by the Aldefluor assay according to the manufacturer’s protocol (Stem Cell Technologies). In our test, cell concentration was 2 × 10^5^ cells/mL and incubation time at 37 ℃ was 40 min. ALDH^high^ and ALDH^low^ cell lines were separated based on fluorescence signals of these cells. *N*,*N*-diethylaminobenzaldehyde, an ALDH inhibitor, was used as negative control to determine Aldefluor-positive cells.

### Quantitative reverse transcription-polymerase chain reaction (qRT-PCR)

Total RNA was extracted using RNeasy Mini Kit (Qiagen) according to the manufacturer’s instructions. RNA intensity was assessed using 2100 Bioanalyzer (Agilent Technologies). Expression levels of target genes were analyzed by ABI 7500 Real-time PCR System. Transcript levels of GAPDH gene was used for normalization. The relative mRNA expression levels of target genes were calculated by comparative CT method^17^. The primer sequences for various human genes used in this study were listed in Supplementary Table 1.

### Transient transfection of GSCs with siRNA

One negative control and three ALDH1A3 siRNAs (Homo-1485, Homo-953, and Homo-1725) were purchased from GenePharma (Suzhou, China). Then GBM cell lines were transfected with siRNA using lipofectamine2000, according to the instructions exactly. Growth medium was changed 6 h after transfection. The silencing efficiency was verified by qRT-PCR and western blotting 48 h after transfection.

### Lentivirus vectors infection

Lentiviral vectors expressing non-target shRNA and two ALDH1A3-targeted shRNAs (shALDH1A3_1, clone name: NM_000693,31379–1; shALDH1A3_2, clone name: NM_000693,31380–1) were obtained from Genechem (Shanghai, China). Full-length ALDH1A3 lentiviral vector (Lenti-ALDH1A3, clone name: NM_000693,15083–1) was used to enhance ALDH1A3 expression. Infection of lentivirus was performed according to the manufacturer’s protocol. Stable transfection was verified by Green Fluorescent Protein weekly.

### Transwell invasion assay

Equal numbers of GBM cell lines were seeded in abovementioned serum-free DMEM in upper chambers with matrigel coating (Costar), with the lower chambers containing DMEM with 10% FBS. After 24 h incubation at 37 ℃, the invaded cells present on the underside of the transwell membrane were crystal violet stained and counted by microscopy (Zeiss).

### Neurosphere-forming assay

Neurosphere-forming assay was performed in low attachment 96-well plates (Corning). GBM cell lines were dissociated into single cells and counted by FCM flow cytometer (Millipore). Then cells were seeded into 96-well plates containing 100 μL serum-free medium at a density of 100 cells per well. Large enough (diameter > 100 μm for LN229 and >50 μm for U251) neurosphere numbers were counted at 10 days using microscope (Zeiss).

### Glycolysis cell-based assay

Seahorse Stress Kit (Agilent) was used to measure the glycolytic activity according to the manufacturer’s instructions. A total of 10^4^ cells were seeded in 24-well Seahorse plates and cultured overnight in a CO_2_ incubator at 37 ℃. The following day, cells were cultured in XF base media supplemented with 1 mM glutamine (pH 7.4) at 37 °C for 1 h in a custom incubator without CO_2_. Assay buffer and reaction solution were prepared based on the manufacturer’s kit reagents. The extracellular acidification rate was analyzed using the XF-Analyzer (Seahorse Bioscience). After the above test, cell number of each well was measured by MTT test.

### In vivo xenograft growth

All animal experiments were performed at the animal laboratory of Beijing Neurosurgical Institute according to National Institutes of Health guidelines. Dissociated GBM cell lines (2 × 10^5^ cells in 5 μL of PBS) were stereotactically injected into the right hemisphere of nude mice. Tumor growth was monitored by 7 T magnetic resonance imaging. Fifty days later, mice were sacrificed and brains were obtained by surgical operation.

### Immunohistochemical and immunofluorescent staining

Paraffin-embedded tissues with complete clinical information were obtained from CGGA Tissue Bank. This study was approved by the institutional review boards, and written informed consent was obtained from each patient. Antibodies for immunohistochemical staining were ALDH1A3 antibody (1:100, Abcam), CD44 antibody (1:100, Cell signal), andSOX2 antibody (1:100, Abcam). Antibodies for immunofluorescent staining were ALDH1A3 antibody (1:100, Abcam), CD44 antibody (1:1000, Cell signal), and SOX2 antibody (1:200, Abcam).

### Establishment of molecular classification scheme

Dimension reduction analysis of eight GSC phenotype-related genes (ALDH1A3, BCL2A1, CD44, LYN, NOTCH1, OLIG2, PROM1, and SOX2) were performed by LASSO-COX method in CGGA Database. The most representative genes and the corresponding coefficients (ALDH1A3: 0.063605344, BCL2A1: 0.018525783, CD44: 0.157272965 and OLIG2: −0.085426518) were identified and selected as the candidate genes to develop the mesenchymal differentiation classification scheme. Finally, to assess the survival prediction value of this 4-gene signature, the risk score for each patient was calculated as previously described^18^.

### Nomogram analysis

Nomogram analysis was performed by *rms* package in software environment R (version 3.4.1). The survival rate of GBM patients could be exactly predicted by total points, sum points of every prognostic factors. Calibrate curves validated the accuracy of the survival rate prediction.

### Statistical analysis

All statistical computations were performed with the statistical software environment R (version 3.4.1), SPSS statistical package (version 19), GraphPad Prism (version 7), and Microsoft Excel 2016. For all statistical methods, *p* < 0.05 was considered significant.

## Results

### ALDH1A3 associates with mesenchymal differentiation of GBM in Eastern and Western world patients

**Fig. 1:**
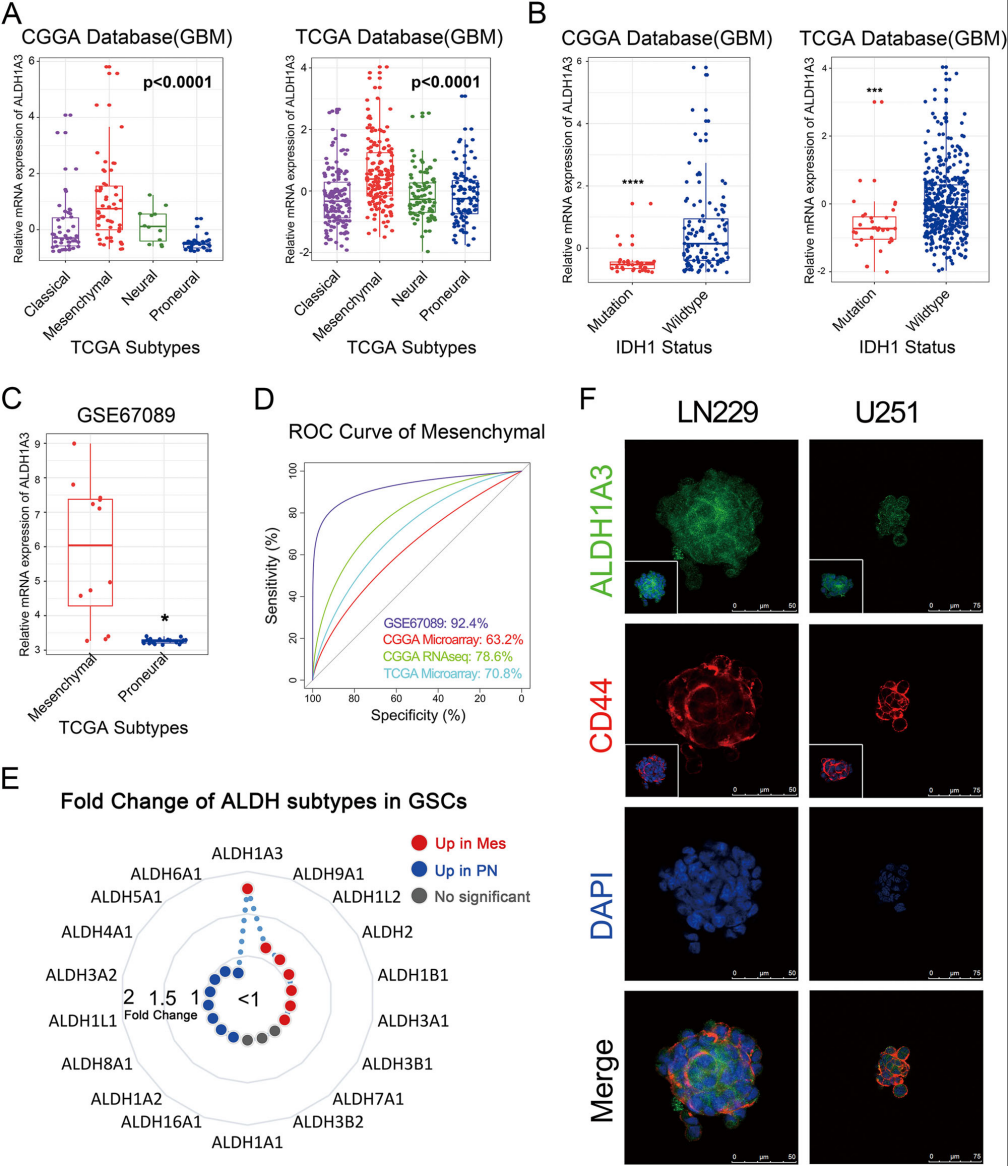
Expression pattern of ALDH1A3 in GBM samples and GSCs. **a** Among TCGA transcriptome subtypes, ALDH1A3 was enriched in mesenchymal subtype in GBM patients. **b** ALDH1A3 was highly expressed in IDH1 wild-type GBM patients. **c** In GSE67089 database, ALDH1A3 was enriched in mesenchymal subtype GSCs. **d** Receiver operator characteristic curves showed that ALDH1A3 had higher sensitivity and specificity to predict MES subtype in GSCs than in GBMs. **e** Among 19 isoforms of ALDH, ALDH1A3 was the most upregulated isoform in MES GSCs in significance analysis of microarrays (SAM) algorithm. **f** Immunofluorescence staining of GSCs showed co-localization expression of ALDH1A3 and CD44. *p < 0.05, ***p < 0.001, ****p < 0.0001

Expression pattern of ALDH1A3 was assessed in 674 GBM patients from CGGA and TCGA databases, revealing a strong overexpression in the MES subtype of GBMs (Fig. 1a). We also noticed that ALDH1A3 expression was significantly reduced in tumors harboring isocitrate dehydrogenase 1 (IDH1) mutation (Fig. 1b). In addition, ALDH1A3 showed higher expression specificity in mesenchymal subtype in GSCs than GBM patients while assessing GSE67089 database (Fig. 1c, d). Amongst all ALDH isoforms, ALDH1A3 was most significantly upregulated in MES subtype GSCs (Fig. 1e). Co-expression of well-known phenotypic markers is a common method for identifying a new phenotypic marker^19^. Therefore, co-expression of ALDH1A3 and a well-known MES differentiation marker CD44 in glioma cells reveals its potential as a MES marker (Fig. 1f).

### ALDH1A3 serves as the key contributor to ALDH activity in glioma cells and represents a target to inhibit tumor cell clonogenicity in vitro

**Fig. 2:**
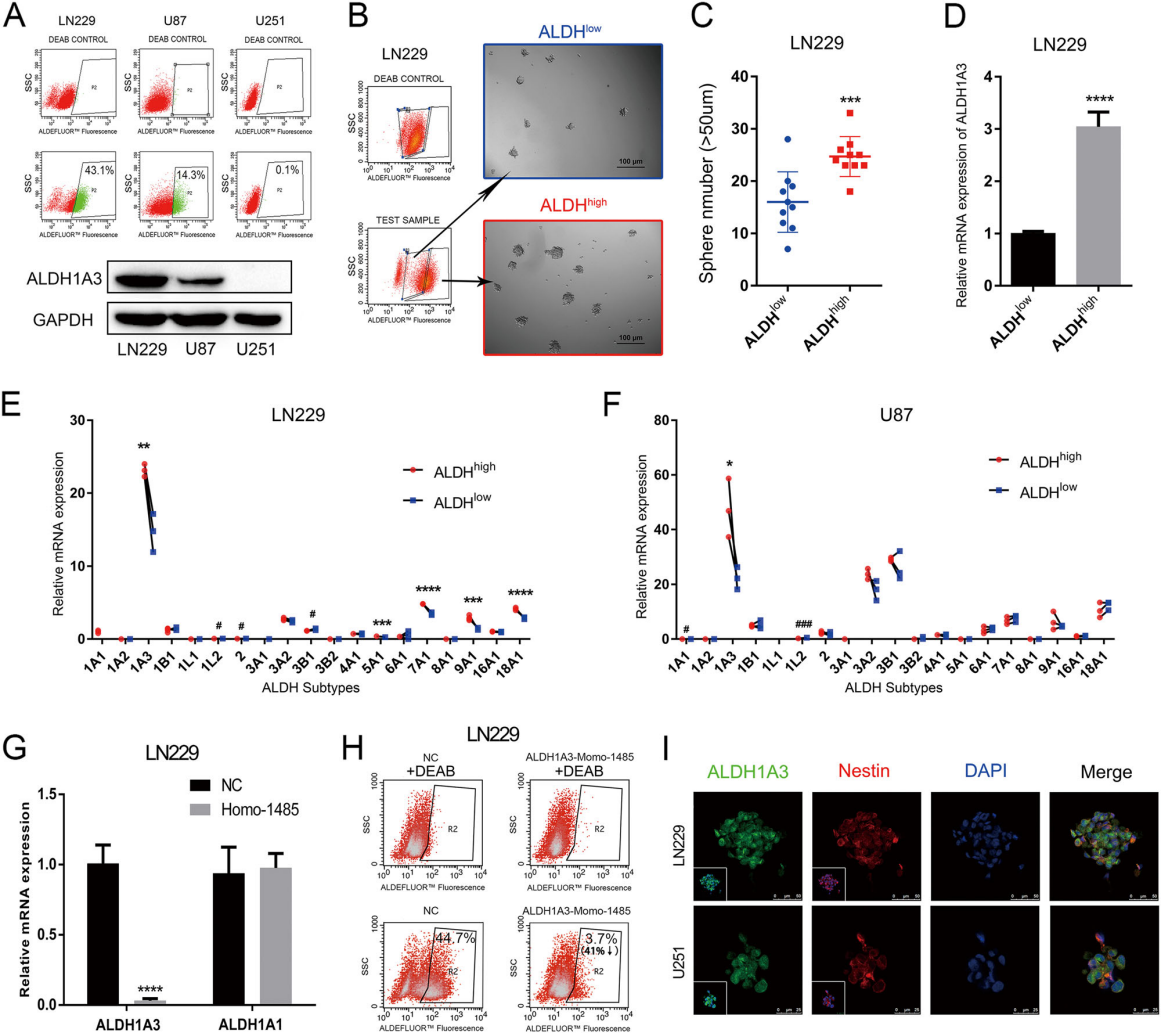
ALDH1A3 is responsible for ALDH enzyme activity in GBMs. **a** Flow cytometry revealed that the protein expression of ALDH1A3 was positively correlated with the enzyme activity of ALDH. **b**, **c** Fluorescence-activated cell sorting sorted ALDH^high^ GBM cells formed more and larger neurospheres than ALDH^low^ ones. ***p < 0.001. **d** Compared to ALDH^low^ LN229, expression of ALDH1A3 was much higher in ALDH^high^ ones. ****p < 0.0001. **e**, **f** ALDH1A3 showed the highest expression and the greatest changes among ALDH^high^ and ALDH^low^ GBM cells. **g** ALDH1A3-targeted siRNA effectively inhibited the expression of ALDH1A3, with no significant changes in ALDH1A1. ****p < 0.0001. **h** Aldefluor assay indicated that ALDH1A3 inhibition caused dramatically decrease of ALDH activity (44.7% to 3.7%). **i** Immunofluorescence stain showed colocalization of ALDH1A3 and Nestin in LN229 and U251

In a baseline activity screen of in vitro glioma models, we identified that GBM cell lines with high ALDH enzyme activity positively correlate with ALDH1A3 protein overexpression (Fig. 2a). When separating ALDH^high^ vs. ALDH^low^ GBM cells, we found ALDH1A3 significantly upregulated in highly clonogenic ALDH^high^ cells compared to ALDH^low^ counterparts (Fig. 2b–d). Of note, amongst all isoforms ALDH1A3 was most strongly activated in both GBM cell lines and also was the isoform which most severely reduced in low clonogenic ALDH^low^ cells (Fig. 2e, f). Strikingly, genetic blockade of ALDH1A3 (Fig. 2g) caused dramatic decrease in total ALDH activity (Fig. 2h and Supplementary Fig. 1). Moreover, ALDH1A3 expressed clones also showed prominent neural stem cell marker-Nestin (Fig. 1i). We summarize that ALDH1A3 is a therapeutic target to diminish the ALDH activity and represents the stemness of tumor cells in vitro.

### ALDH1A3 is an activator of mesenchymal transformation in GBM

**Fig. 3:**
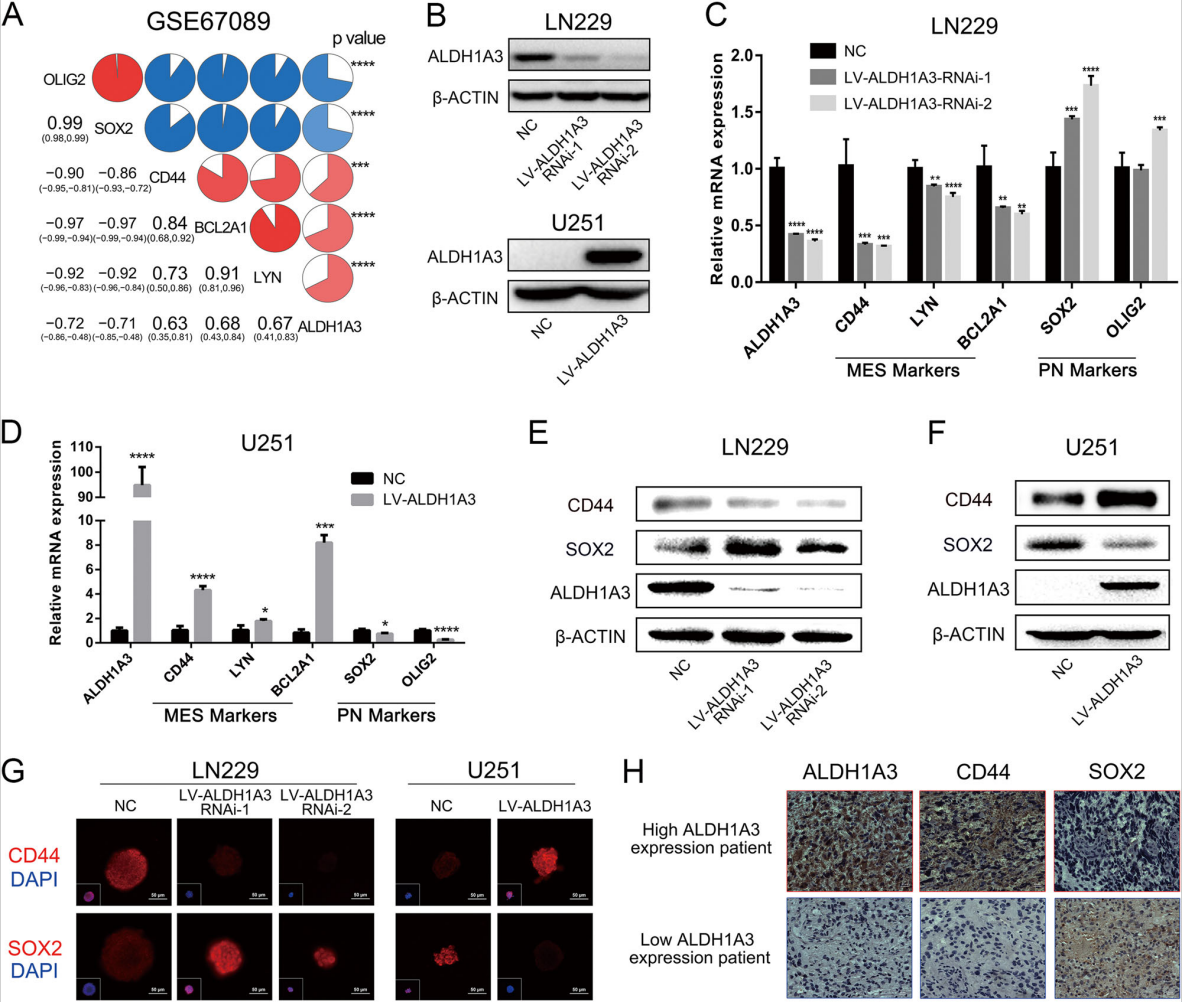
ALDH1A3 is a driver for PMT in GBMs. **a** In GSCs database, ALDH1A3 was positively correlated with MES signatures and negatively correlated with PN signatures. ***p < 0.001, ****p < 0.0001 analyzed by Pearson correlation analysis. Red represents positive correlation and blue represents negative correlation. **b** Expression of ALDH1A3 was dramatically decreased or over-expressed by lentiviruses infection. **c** Effects of ALDH1A3 inhibition by LV-ALDH1A3-RNAi-1 and LV-ALDH1A3-RNAi-2 on GSCs phenotype markers. **p < 0.01, ***p < 0.001, ****p < 0.0001. **d** Effects of ALDH1A3 overexpression by LV-ALDH1A3 on GSCs phenotype markers. *p < 0.05, ***p < 0.001, ****p < 0.0001. **e**, **f** Western blotting verified the effects of ALDH1A3 inhibition or overexpression on MES marker CD44 and PN marker SOX2. **g** Immunofluorescence staining verified the effects of ALDH1A3 inhibition or overexpression on MES marker CD44 and PN marker SOX2. (**h**) Immunohistochemical staining of untreated GBM specimens showed that ALDH1A3 expression strongly correlated with CD44 expression, whereas the staining of ALDH1A3 and SOX2 were mutually exclusive

Mesenchymal transformation has emerged as cardinal driver for progression in malignant tumors^6^. Reminiscent of epithelial to mesenchymal transformation, differentiation from proneural to mesenchymal identity (proneural to mesenchymal transformation, PMT) can cause malignant progression of GBM^16^. Consistent with previous classic study, OLIG2 and SOX2 were considered as proneural (PN) markers, whereas CD44, BCL2A1, and LYN were used as MES markers^16^. Pearson correlation analysis of GSCs microarray database revealed that ALDH1A3 was positively correlated with all MES markers and negatively correlated with all PN markers (Fig. 3a). This result was further validated in GBM samples from CGGA and TCGA databases (Supplementary Fig. 2). Furthermore, we created GBM cells with genetically induced inhibition and overexpression of ALDH1A3 (Fig. 3b and Supplementary Fig. 3). In concordance to the clinical data, modulation of ALDH1A3 caused proportional shifts in PMT as assessed via quantifying the expression of named lineage specific markers (Fig. 3c–g). Importantly, in untreated GBM samples, we also found that high expression of ALDH1A3 was positively associated with the expression of CD44 and negatively associated with SOX2 (Fig. 3h). Taken together, we showed the key role of ALDH1A3 in promoting PMT in GBM cells and patients from multiple aspects.

### ALDH1A3 inhibition diminishes cell invasion, cell proliferation, glycolysis, and reduces tumor growth in vivo

**Fig. 4:**
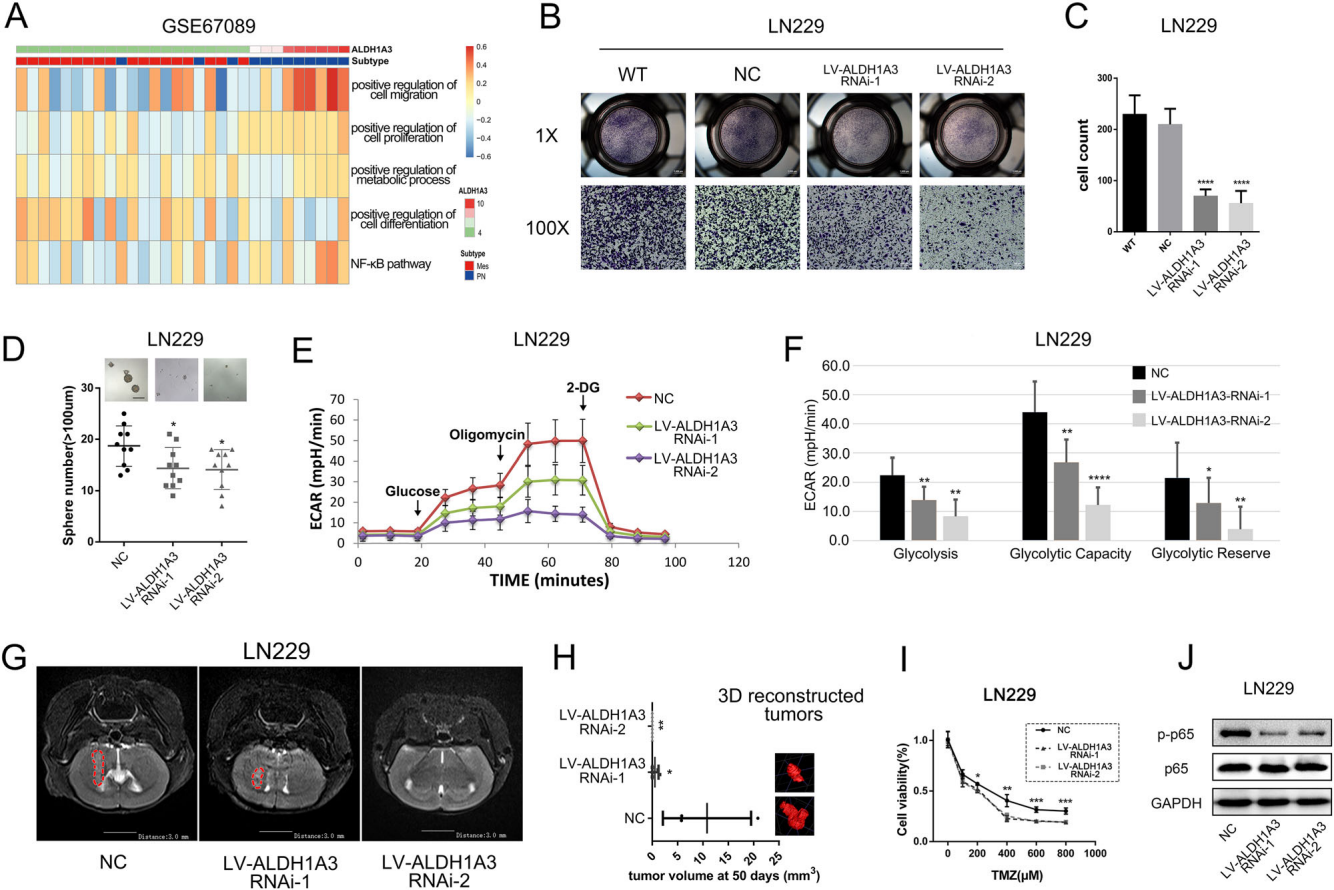
The oncogenic potential of ALDH1A3 in regulating GBM stemness, invasion and tumor growth in vitro and in vivo. **a** GSVA analysis showed that ALDH1A3 expression were closely associated with cell migration, cell proliferation, metabolic process, cell differentiation, and NF-κB pathway. **b**, **c** Transwell assay showed that ALDH1A3 inhibition induced obviously decrease of its invasion ability in GSCs. ****p < 0.0001. **d** Neurosphere formation experiments showed a strong correlation between ALDH1A3 expression and self-renewal ability. *p < 0.05. **e**, **f** ALDH1A3 silence markedly inhibited glycolysis, glycolytic capacity and glycolytic reserve of GSCs. *p < 0.05, **p < 0.01, ****p < 0.0001. **g**, **h** ALDH1A3 inhibition lead to inhibition of tumorigenic ability significantly in orthotopic transplanted mice. *p < 0.05, **p < 0.01. The tumor border of mice was delineated by an experienced neuroradiologist on T2-weighted images using the IKT-SNAP software (Version 3.6.0), and the tumor volume was quantified with the module implemented in IKT-SNAP. **i** Inhibition of ALDH1A3 in LN229 increased the sensitivity of GSCs to chemotherapy drug-TMZ. This sensitization effect was more significant with increasing TMZ concentration. *p < 0.05, **p < 0.01, ***p < 0.001. **j** Western blotting showed ALDH1A3 inhibition reduced phosphorylated p65 with no significant change in p65

We further explored the biological process of ALDH1A3 in GSCs. Gene Set Variation Analysis (GSVA) of GSE67089 database indicated that ALDH1A3 expression was positively associated with cell migration, cell proliferation, metabolic process and NF-κB pathway, and negatively associated with cell differentiation (Fig. 4a). In functional studies using cells with genetic blocked ALDH1A3 expression, we noticed a strong decrease in cellular invasion and neurosphere formation as compared to cells carrying the control vector (Fig. 4b–d). On the contrary, ALDH1A3 overexpression resulted in significantly increased invasion abilities (Supplementary Fig. 4A, 4B and 4C). In concordance to previous reports, we noticed a direct effect on glycolysis parameters in dependency of ALDH1A3 activation using live cell metabolism (Fig. 4e, f, Supplementary Fig. 4D and 4E). Importantly, ALDH1A3 blockade could significantly reduce tumor growth in vivo (Fig. 4g, h). In vitro experiments also confirmed that reducing the expression of ALDH1A3 could increase the sensitivity of GBM cells to temozolomide (TMZ) chemotherapy (Fig. 4i). As a mechanistic explanation of the observed phenotypes, we hypothesized that ALDH1A3 functions through NF-κB pathway as phosphorylated form of p65, a known upstream regulator of the pathway, was reduced when inhibiting ALDH1A3 (Fig. 4j). As we all know, these biological functions are closely related to the MES phenotype in many cancers. Therefore, our results further confirmed the role of ALDH1A3 in PMT promoting from the perspective of biological functions in vitro and in vivo.

### A novel molecular classification scheme based on PMT markers precisely predicts clinical prognosis of GBM patients

**Fig. 5:**
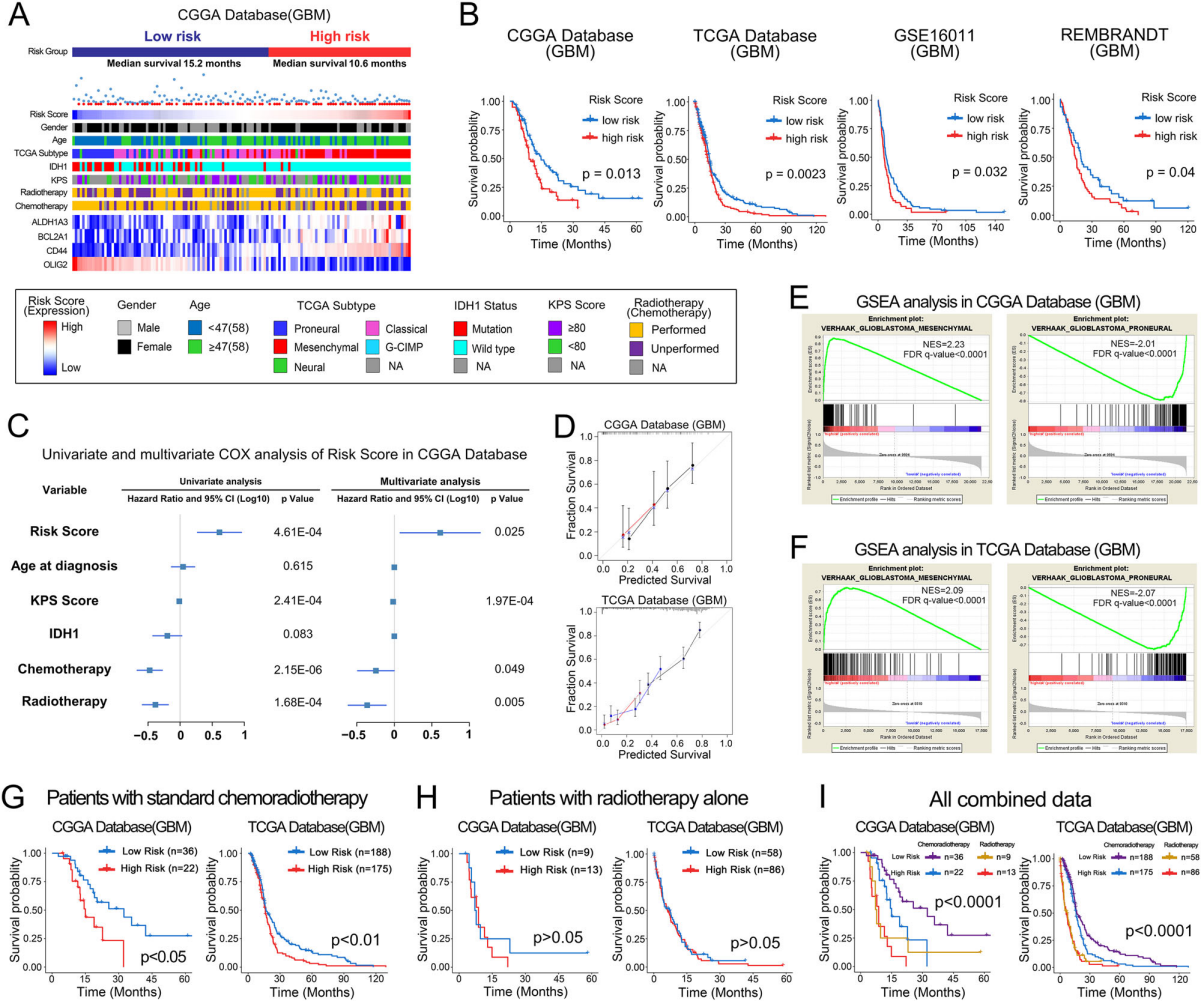
The novel molecular classification scheme based on ALDH1A3-derived PMT genes. **a** Landscape of clinical features in different molecular classification groups in GBM patients of CGGA database. **b** Kaplan–Meier survival analysis showed that patients with high risk of the molecular classification scheme conferred a worse prognosis in GBM patients of CGGA, TCGA, GSE16011, and REMBRANDT databases. **c** Univariate and multivariate COX analysis showed that the molecular classification scheme was an independent prognostic factor in GBM patients of CGGA database. **d** The molecular classification scheme could accurately predict survival rate of GBM patients of CGGA and TCGA databases. **e**, **f** GSEA showed that GBM patients with high risk score were highly enriched in MES subtype and low risk patients were most enriched in PN subtype. **g**–**i** The Kaplan–Meier estimates of overall survival in GBM patients from CGGA and TCGA database illustrate survival according to risk-score and postoperative treatment strategies for the standard chemoradiotherapy subgroup (**g**), the radiotherapy alone subgroup (**h**), and all patients combined (**i**)

In prognosis prediction, molecular classification scheme is more stable than single gene expression profiling^18,20^. Therefore, we established a novel molecular classification scheme centered on PMT markers in 138 GBM patients. According to the risk-score analysis of this scheme, all GBM patients could be divided into high-risk and low-risk subgroups. The relationship between the risk score and clinical characteristics of GBM patients from CGGA and TCGA database was showed in Fig. 5a and Supplementary Fig. 5. Kaplan–Meier survival analysis revealed that GBM patients classified to high-risk group conferred worse clinical outcome in experimental (CGGA) and three validation databases (TCGA, GSE16011 and REMBRANDT) (Fig. 5b). Furthermore, univariate and multivariate COX regression analysis showed that the risk score was an independent prognostic factor after adjusting for other prognostic factors, such as patient age, Karnofsky performance score, IDH1 mutation status, chemotherapy and radiotherapy (Fig. 5c and Supplementary Fig. 6). In order to evaluate the predictive power of this classification scheme, a prognostic nomogram was established to predict 1-, 2- and 3-year survival rates of GBM patients based on risk score and other prognostic clinical characteristics (Supplementary Fig. 7). Importantly, this molecular classification scheme showed high predictive accuracy in GBM patients’ survival prediction (Fig. 5d).

### The classification scheme predicts the therapeutic benefits for GBM patients with standard chemoradiotherapy but not for patients with radiotherapy alone

Gene set enrichment analysis (GSEA) indicated that GBM patients with high risk score were more likely to be grouped into MES subtype while low-risk score patients showing a greater tendency to PN subtype (Fig. 5e, f). It is known that PMT was the main reason for postoperative radiotherapy and chemotherapy resistance in GBM^21,22^. Therefore, we reevaluated the predictive effect of the classification scheme in GBM patient subgroups with different postoperative therapeutic strategies. Interestingly, in GBM patients treated with postoperative standard chemoradiotherapy, high risk group displayed significantly shorter survival time (Fig. 5g). However, for GBM patients receiving post-operative radiation alone, this classification scheme showed not informative for survival prediction (Fig. 5h). This interesting result indicated that this classification scheme could predict the therapeutic benefits for GBM patients with standard chemoradiotherapy but not for patients with radiotherapy alone. Then, we combined all the patient subgroups together and observed that GBM patients who had low-risk scores and received standard chemoradiotherapy would have the best outcome (32.33 months in CGGA and 16.9 months in TCGA), and patients with high-risk scores and radiotherapy alone had the shortest survival (8.77 months in CGGA and 6.23 months in TCGA) (Fig. 5i).

## Discussion

ALDH has emerged as a critical target in cancer therapy with a particular high effectivity to eradicate cancer stem-like cells^9,23,24^. Particularly the isoform ALDH1A3 has been shown to serve as very powerful therapeutic target^14,25^. In GBM, ALDH1A3 has been described to associate with the mesenchymal subtype of the tumor^16,26,27^. Several upstream and downstream mediators of the malignant properties of ALDH1A3 in glioma cells have been identified^16,26,28^. However, we unexpectedly found that until now there was no direct evidence which proved that it was ALDH1A3 that was a major contributor to ALDH activity and also a key driver in triggering mesenchymal transformation in GBM, although there were some studies that might mention this. This is of particular high clinical interest as mesenchymal transdifferentation is considered to intertwine with stemness and a main cause of tumor progression^9,28^.

Our study not only recapitulated ALDH1A3 association to the mesenchymal lineage of GBM, but also revealed its cardinal role in inducing the MES differentiation. Firstly, we not only reproduced the high enrichment of ALDH1A3 in the MES in GSCs and GBM, but also further revealed its special expression pattern in different IDH mutation status and its different MES enrich specificity with other ALDH isoforms. These results have further complemented previous studies, and more importantly, it highlighted the special status of ALDH1A3 in ALDH, which was not mentioned before. Secondly, ALDH1A3 was proved to be responsible for the total ALDH activity by directly gene knocking down. Knowing that the ALDH was useful in the identification of stem cell populations, this result provided a theoretical basis for more accurate stem cell-targeted therapy in the future. Thirdly, we provided further evidences that ALDH1A3 was a key driver of mesenchymal differentiation. These results seem to lack sufficient innovation and no surprise, but it is the only comprehensive study of ALDH1A3’s functions in glioma. Although Mao’s article has studied the role of ALDH in radiotherapy resistance in GSC, our study more clearly clarified the comprehensive role of ALDH1A3^16^. The study of phenotypes and biological functions laid the foundation for the future in-depth study of ALDH1A3. Last but not the least, our data presented the hitherto largest clinical assessment (*n* = 674) of ALDH1A3 in GBM patients from both the Western and Eastern world. Our analyses revealed that molecular classification scheme based on PMT markers could accurately predict the clinical prognosis and guide treatment strategies of GBM patients, which achieved the clinical transformation of PMT functional studies.

This was of great clinical importance as a portion of GBM patients have favorable prognosis and an unequivocal identification of those patients would not only enhance the personal life quality but may also improve management and therapy decision making for those patients^29–31^. Therefore, stable prediction models that can accurately predict the prognosis of GBM patients are highly warranted. In our previous study, we indicated that hypermethylation of ALDH1A3 predicted a better prognosis in primary GBM patients^32^. In various tumors of other origin, ALDH1A3 was found to have prognostic value^27,33,34^. Of note, in our experiences in the analysis of clinical data, we found that prognosis prediction based on a single gene transcription or methylation pattern has rather limited predictive power^18,20^. Therefore, given the identified driver role of ALDH1A3 for MES differentiation, we established a novel molecular classification scheme based on ALDH1A3-derived PMT genes in GBM patients. Importantly, this molecular classification scheme possessed extraordinary stable predictive value in the largest cohort of GBM patients (*n* = 932) amongst all databases we could access (CGGA, TCGA, GES16011, and REMBRANDT Databases). In addition to evaluate the patient survival, we found that this classification scheme performed well in therapeutic benefit prediction for GBM patients with postoperative standard chemoradiotherapy. In 2013, Mao’s research team found that high MES GSCs showed highly resistant to radiation therapy^16^. Unfortunately, they did not confirm this finding in patients. Analyzing large samples of clinical data, we found that MES and PN groups of GBM patients showed no difference in the benefit of radiotherapy. Conversely, the prognosis of PN group was significantly better than that of MES group after chemotherapy. Therefore, we speculated that ALDH1A3-derived PMT blocking might effectively improve the chemotherapeutic sensitivity of GBM patients and consequently prolong the overall survival time. Then we designed the in vivo experiment to confirm this hypothesis (Fig. 4i). In future, more evidence is needed to verify this hypothesis.

In conclusion, our integrated study based on large sample databases and experimental techniques further support the hypothesis of clinically evaluation of ALDH1A3 in GBM therapy. Of note, recently the use of a pan-ALDH inhibitor has been shown the promising effect^26^. However, the adverse effects caused by such non-targeted anti-ALDH therapy need to be taken in consideration, as non-malignant neural stem cells are also highly dependent on ALDH activity^35^. We therefore suggest investigate the therapeutic potential of ALDH1A3-specific targeting compound in the context of brain tumor^12^. The development and application of such a target drug need to make hard exploration and efforts. At least we can now apply the molecular classification scheme to clinical practice as soon as possible so that patients can benefit early.

## Electronic supplementary material

Supplementary Figure 1

Supplementary Figure 2

Supplementary Figure 3

Supplementary Figure 4

Supplementary Figure 5

Supplementary Figure 6

Supplementary Figure 7

Supplementary Table 1

Supplementary figure legends
