# Supplementary figures and images for: ALDH1A3 induces mesenchymal differentiation and serves as a predictor for survival in glioblastoma
Source: Cell Death Dis. 2018 Dec 11;9(12):1190. doi: 10.1038/s41419-018-1232-3 (PMC6290011; doi:10.1038/s41419-018-1232-3)

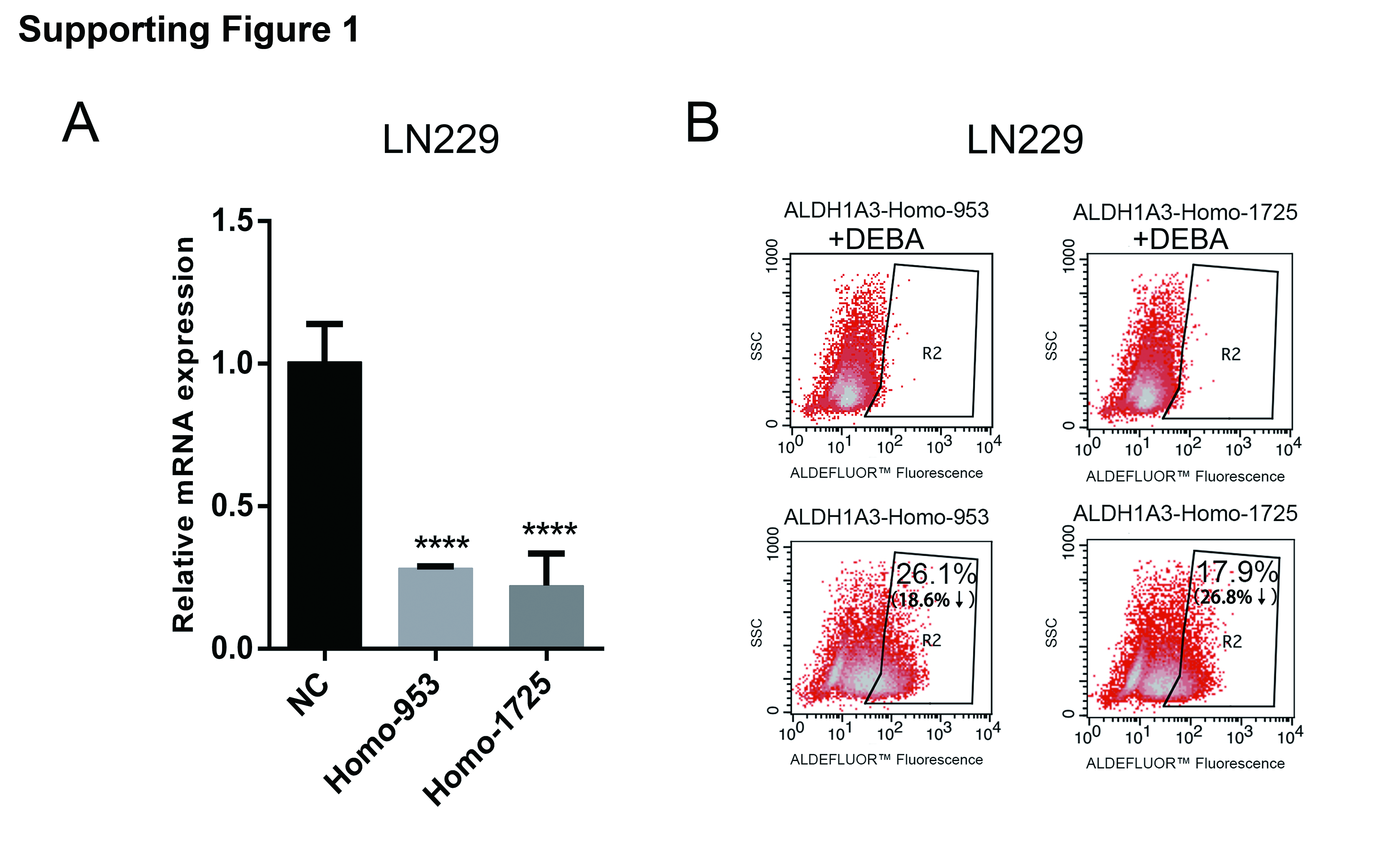

Supplement: Supplementary file 1 — Supplementary Figure 1 [file 41419_2018_1232_MOESM1_ESM.tif]

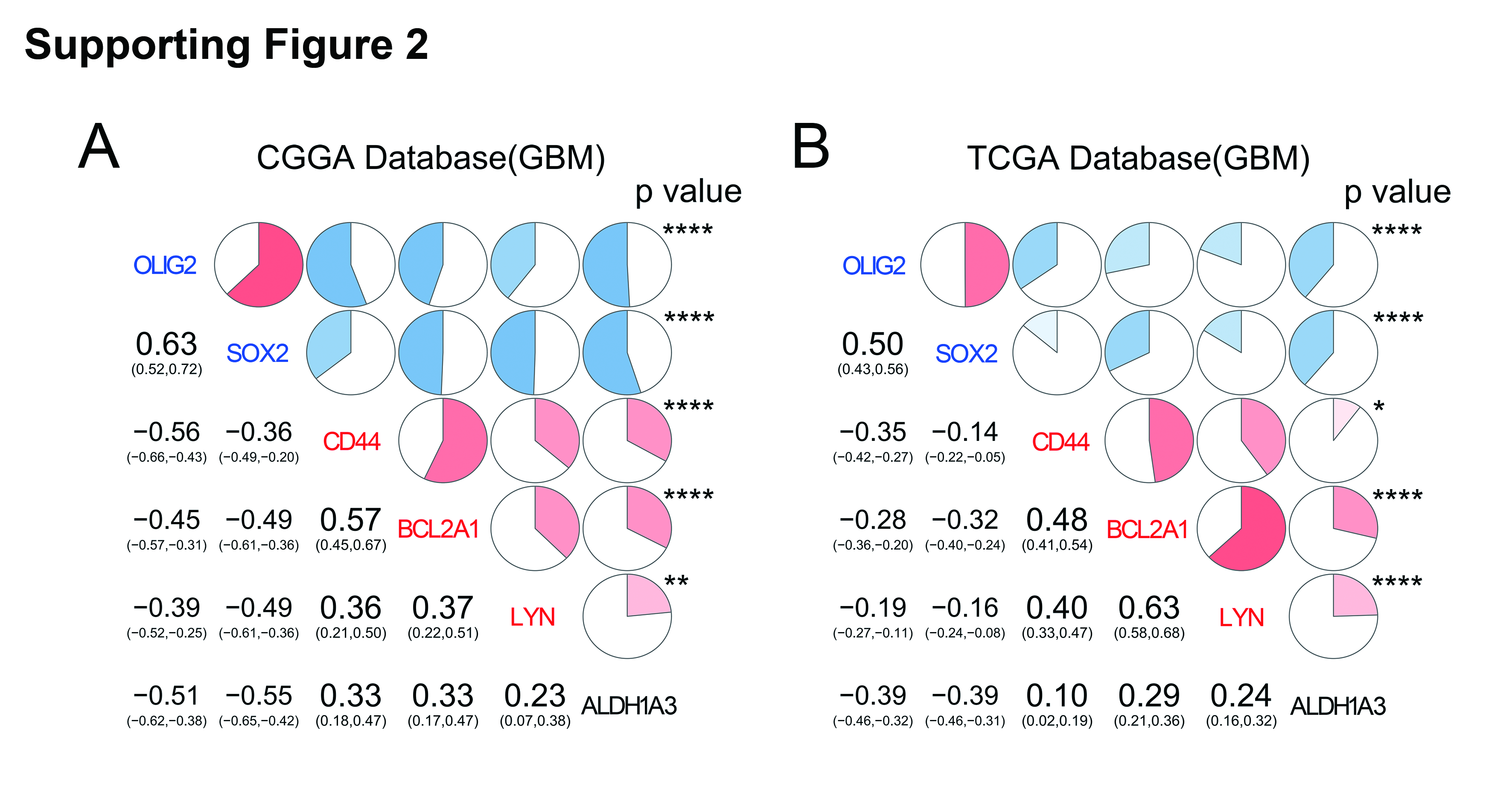

Supplement: Supplementary file 2 — Supplementary Figure 2 [file 41419_2018_1232_MOESM2_ESM.tif]

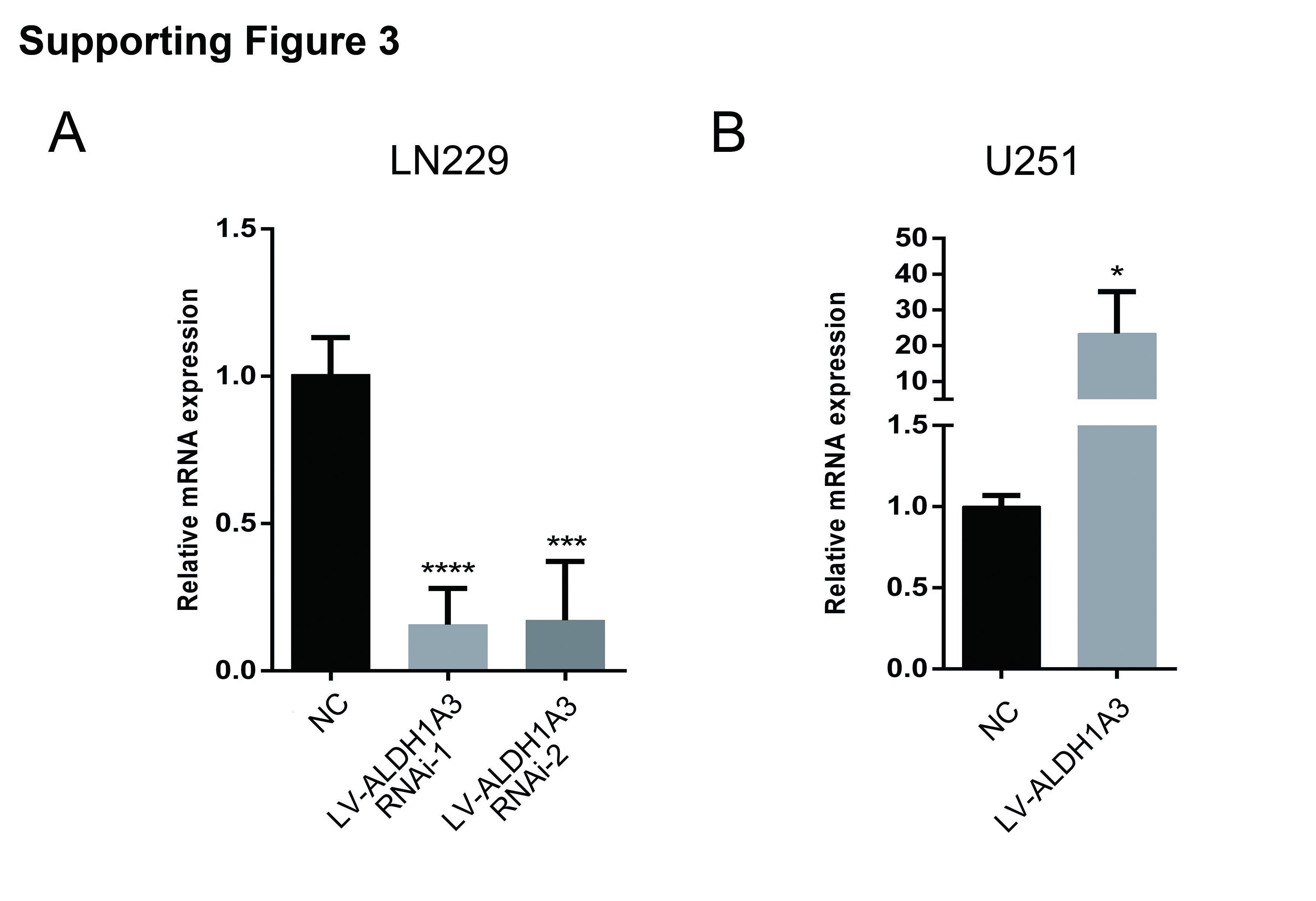

Supplement: Supplementary file 3 — Supplementary Figure 3 [file 41419_2018_1232_MOESM3_ESM.tif]

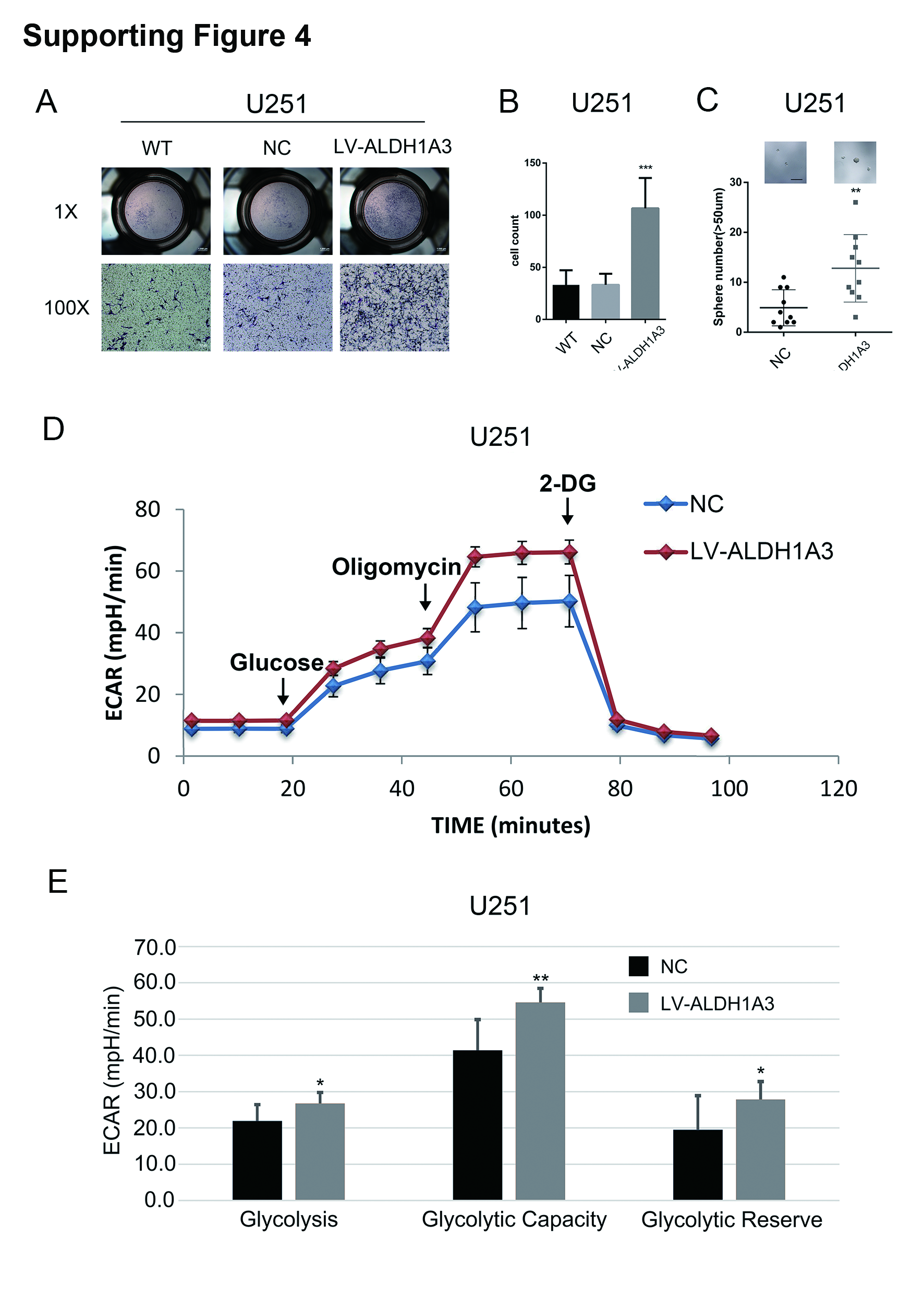

Supplement: Supplementary file 4 — Supplementary Figure 4 [file 41419_2018_1232_MOESM4_ESM.tif]

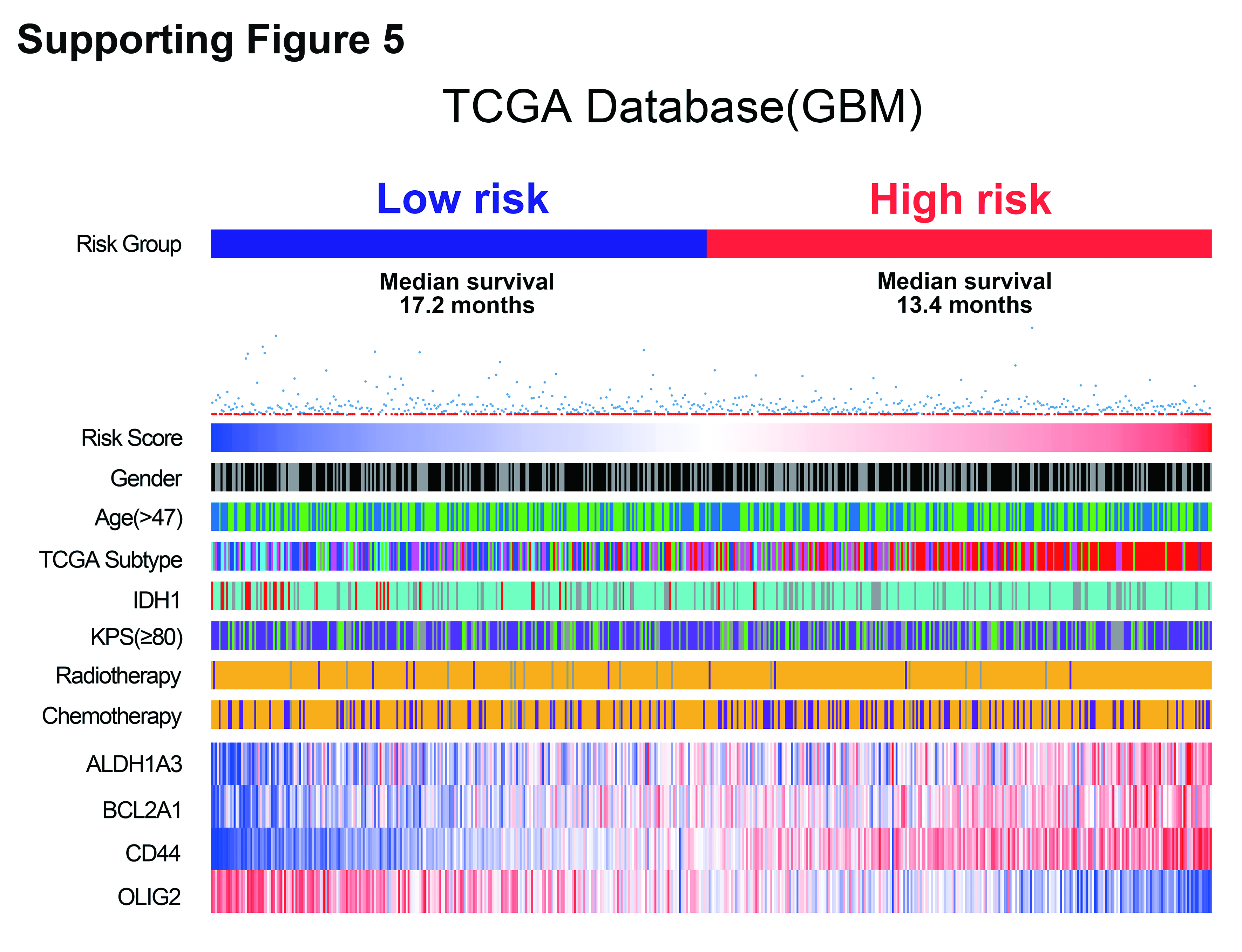

Supplement: Supplementary file 5 — Supplementary Figure 5 [file 41419_2018_1232_MOESM5_ESM.tif]

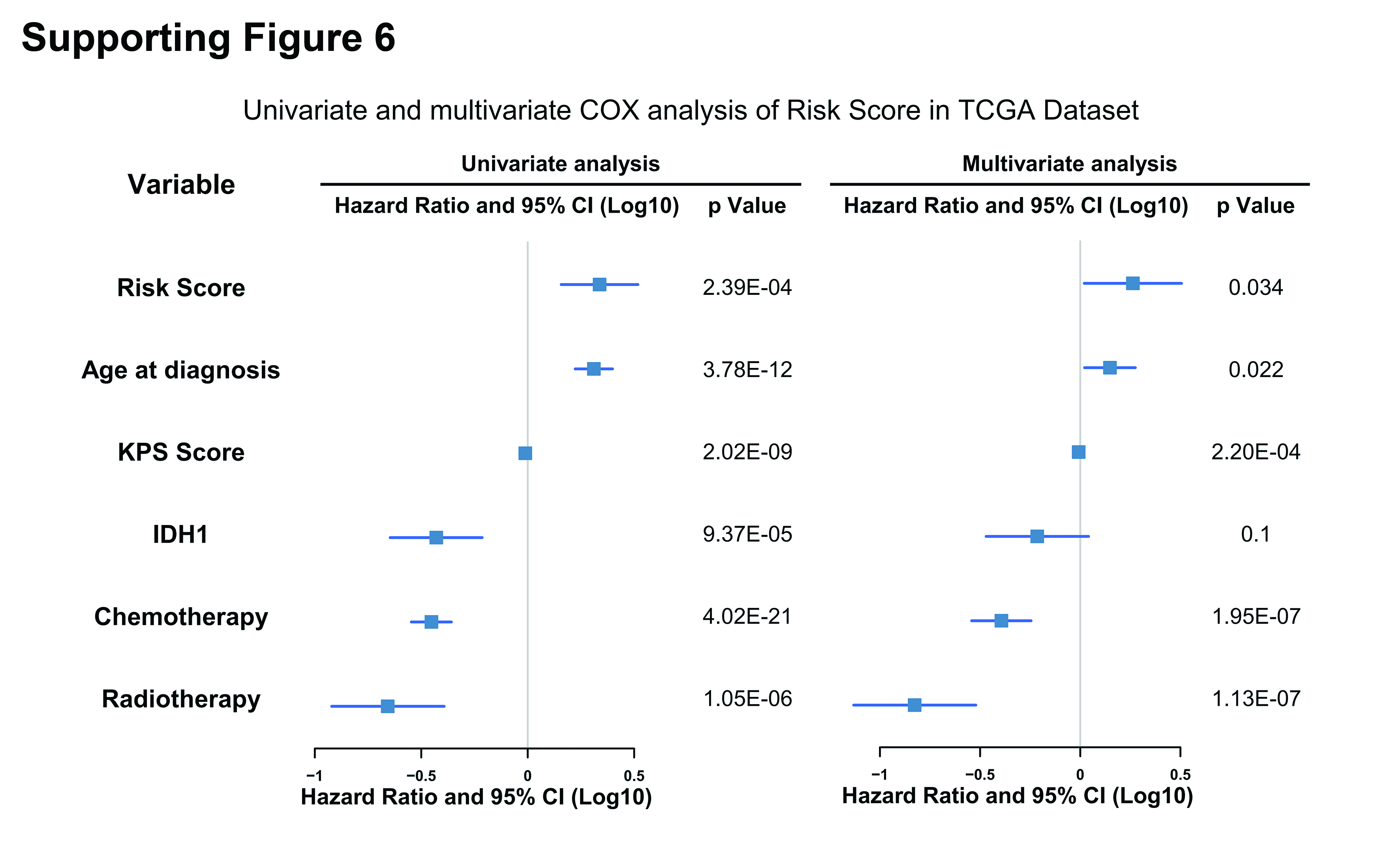

Supplement: Supplementary file 6 — Supplementary Figure 6 [file 41419_2018_1232_MOESM6_ESM.tif]

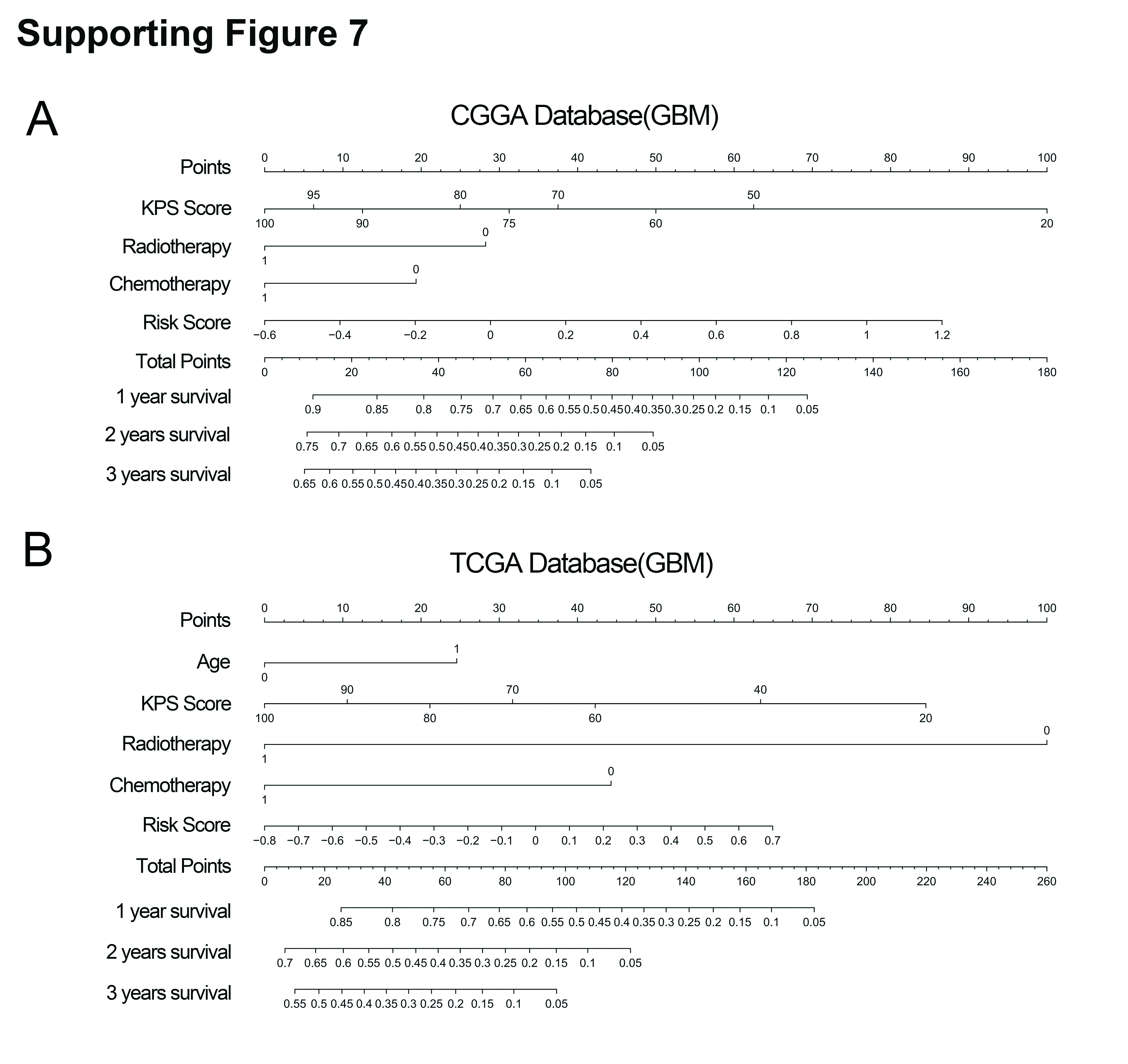

Supplement: Supplementary file 7 — Supplementary Figure 7 [file 41419_2018_1232_MOESM7_ESM.tif]
