# Supplementary material for: ALDH1A3 induces mesenchymal differentiation and serves as a predictor for survival in glioblastoma
Source: Cell Death Dis. 2018 Dec 11;9(12):1190. doi: 10.1038/s41419-018-1232-3 (PMC6290011; doi:10.1038/s41419-018-1232-3)
Supplement: Supplementary file 8 — Supplementary Table 1 [file 41419_2018_1232_MOESM8_ESM.docx]

**Supplementary Table 1 The primer sequences of PMT genes**

| Gene | Forward primer (5’-> 3’) | Reverse primer (5’-> 3’) |
| --- | --- | --- |
|  |  |  |
| GAPDH | GGAGCGAGATCCCTCCAAAAT | GGCTGTTGTCATACTTCTCATGG |
| ALDH1A3 | TGAATGGCACGAATCCAAGAG | CACGTCGGGCTTATCTCCT |
| SOX2 | ACCGGCGGCAACCAGAAGAACAG | GCGCCGCGGCCGGTATTTAT |
| CD44 | CCCAGATGGAGAAAGCTCTG | ACTTGGCTTTCTGTCCTCCA |
| CD133 | ACTCCCATAAAGCTGGACCC | TCAATTTTGGATTCATATGCCTT |
| OLIG2 | CTCCTCAAATCGCATCCAGA | AGAAAAAGGTCATCGGGCTC |
| NOTCH1 | GAGGCGTGGCAGACTATGC | CTTGTACTCCGTCAGCGTGA |
| LYN | CTGAACTCAAGTCACCGTGG | TCCATCGTCACTCAAGCTGT |
| BCL2A1 | ATGGATAAGGCAAAACGGAG | TGGAGTGTCCTTTCTGGTCA |
| WT1 | TTAAAGGGAGTTGCTGCTGG | GACACCGTGCGTGTGTATTC |
