# Supplementary material for: ALDH1A3 induces mesenchymal differentiation and serves as a predictor for survival in glioblastoma
Source: Cell Death Dis. 2018 Dec 11;9(12):1190. doi: 10.1038/s41419-018-1232-3 (PMC6290011; doi:10.1038/s41419-018-1232-3)
Supplement: Supplementary file 9 — Supplementary figure legends [file 41419_2018_1232_MOESM9_ESM.docx]

**Supplementary Figure Legends**

**Supplementary Fig. 1** **ALDH1A3 plays an important role in ALDH enzyme activity in GSCs.** (A) Expression of ALDH1A3 was significantly decreased by siRNA transfection. ****p<0.0001. (B) ALDH1A3 inhibition leaded to dramatically decrease of ALDH activity in LN229 by Aldefluor assay.

**Supplementary Fig. 2 The relationship between ALDH1A3 and PMT markers in GBM samples.** (A, B) ALDH1A3 was positively correlated with MES markers and negatively correlated with PN markers in GBM patients of CGGA and TCGA databases. *p<0.05, **p<0.01, ****p<0.0001 analyzed by Pearson correlation analysis. ns means no statistical differences.

**Supplementary Fig. 3 QRT-PCR verification of infection efficiency of lentiviruses.** (A, B) qRT-PCR verified transfection efficiency of lentiviruses in GSCs every two weeks. *p<0.05, ***p<0.001, ****p<0.0001 analyzed.

**Supplementary Fig. 4 ALDH1A3 overexpression increases malignant biological functions of U251 cell line.** (A, B) Transwell assay showed that ALDH1A3 expression enhancement induced obviously increase of invasion ability in U251. ***p<0.001. (C) Self-renewal ability of U251 was positively correlation with ALDH1A3 expression. **p<0.01. (D, E) ALDH1A3 expression enhancement markedly promoted glycolysis, glycolytic capacity and glycolytic reserve of U251. *p<0.05, **p<0.01.

**Supplementary Fig. 5 The relationship between the molecular classification scheme and clinicopathological characteristics in GBM patients.** Landscape of clinical features of GBM patients in different risk score groups in TCGA database.

**Supplementary Fig. 6** **The molecular classification scheme is an independent prognosis factor of GBM patients.** Univariate and multivariate COX analysis of risk score and prognostic clinical factors in GBM patients in TCGA database.

**Supplementary Fig. 7 The molecular classification scheme predicts the survival rate of GBM patients.** Nomograms of the molecular classification scheme and other prognostic clinical factors could predict the 1 year, 2 years and 3 years survival rate of GBM patients in CGGA and TCGA databases.
